# A Plea for the Microscopic Examination of the Blood in the Continued and Remittent Fevers Common to Our Southern States

**Published:** 1903-11

**Authors:** Louis M. Warfield

**Affiliations:** Johns Hopkins, Savannah, Ga.


ATLANTA

### Journal-Record of Medicine.

**Successor to Atlanta Medical and Surgical Journal, Established 1855, and Southern Medical Record, Established 1870.**

VOL. V. NOVEMBER, 1903. NO. 8.

BERNARD WOLFF, M.D., EDITOR, Nos. 319–20 Prudential.

Published Monthly.

M. B. HUTCHINS, M.D., BUSINESS MANAGER, 1007-1008 Century Bldg.

### *ORIGINAL COMMUNICATIONS.*

### A PLEA FOR THE MICROSCOPIC EXAMINATION OF THE BLOOD IN THE CONTINUED AND REMITTENT FEVERS COMMON TO OUR SOUTHERN STATES.

BY LOUIS M. WARFIELD, A.B., M.D. (JOHNS HOPKINS),

SAVANNAH, GA.

That the difficulty of diagnosing certain protracted and continued fevers is still with us in the South a burning question and a serious problem is once again shown by the difference of opinion among physicians in parts of Georgia where epidemics of continued fevers are occurring. Some say it is malaria, some call it typhoid fever, some give it a name "typho-malarial," which means little or nothing, and is a name that, as Johnston says, "leads to confusion and misunderstanding, is a cover for uncertainty and ignorance, and should be discouraged and abandoned." That there is no such hybrid malady as "typho-malaria," or "malario-typhoid," is now a very generally accepted fact. For those who still believe in such a disease entity we can only refer them to standard works on medicine and recent bacteriological researches, as a discussion of such points would go far beyond the limits of this article. One or two facts only will be mentioned. Hare (Medical Complications and Sequelæ of Typhoid Fever, 1899) says that there is no such disease, and quotes Gilman Thompson (*Amer. Jour. Med. Sc.*, August, 1894) and Ewing (*N. Y. Med. Jour.*, February 4, 1899) in suppport of this view. He says (p. 81, *loc. cit.*): "There is no doubt whatever that pure typhoid infection may result in the production of a fever which closely follows the remittent and intermittent malarial types, and which is often associated with so much gastric disturbance and vomiting and so lacking in the more prominent typhoid symptoms usually seen, that the picture of remittent malarial fever is clear, while the true picture of typhoid fever is clouded. Again, there can be no doubt that cases of true malarial infection occur in which the symptoms so closely resemble those of typhoid fever that a purely clinical diagnosis is almost impossible, particularly if an epidemic of typhoid fever is in full swing at the time. Finally, there can also be no doubt that it is possible for the patient to have a double infection with the bacillus of Eberth and the plasmodium of Laveran, in which case, however, the malarial manifestations are usually dwarfed by the typhoid poison, and are only marked at the onset of the enteric fever and at its termination. To this mixed infection the term typho-malarial fever may be correctly applied to indicate not a separate disease but a double infection."

Ewing (*loc. cit.*), in the examination of the blood of 159 cases of typhoid fever at Montauk Point among the soldiers, says that in no case of undoubted typhoid fever were malarial parasites found in the blood. "Many patients whose blood contained numerous parasites were seen in the typhoid state, but there were always some essential symptoms lacking to confirm the diagnosis of typhoid fever, while the subsequent course of the disease demonstrated the purely malarial character of the fever." . . . . "In short, in spite of very painstaking efforts, the attempt to find a case of typhoid fever and active malaria progressing simultaneously was unsuccessful." Such a dogmatic assertion of fact should have much weight in clearing up the haze that surrounds this question, especially in view of the noteworthy fact that the troops quartered at Montauk Point were brought, in most cases, direct from a malaria infested district, and it seems that if the two diseases occurred together frequently that there was the very best soil for the development of the two exciting causes. Yet in spite of that, typhoid fever and malaria did not coexist in the same patient once in 159 cases. Osler (Text-book of Medicine, 1901) has only seen two such double infections in 829 cases of typhoid fever. There can be no question about the rarity of the existence of the two conditions in the same patient. Should a patient with malaria develop typhoid fever, the malarial infection has no effect of any kind on the course of the typhoid fever. It is held in complete abeyance until the convalescence of the enteric fever, when typical chills, fever and sweats may occur and the organisms can be found in the circulating blood.

With such a state of affairs before us how can the general practitioner differentiate the two diseases? As we have seen above, one may ape the other so closely that it is only the subsequent course and the termination that will give us the clue if we rely only on the clinical symptoms for our diagnosis. In the meantime the whole community is in danger. It is not enough to wait several weeks for a diagnosis, or to make a diagnosis on insufficient data. What is needed is an accurate diagnosis in the shortest possible time before the epidemic, if an epidemic, gains headway, in order that intelligent steps may be taken to protect the infected locality. No one means is more of service in such cases than the microscope and a drop of blood. The writer does not underestimate the value of blood cultures, as he has repeatedly been able to make a diagnosis of typhoid fever before the presence of the Widal reaction.* The presence of the typhoid bacilli in the blood is proof positive that the case is typhoid fever. He realizes also the value of the Widal reaction, but in both these methods a bacteriological laboratory must be near at hand. For the busy practitioner these methods are nearly always out of the question, hence we wish to emphasize the value of the microscopic examination of

* See Bull. of The Josephine M. Ayle Clin. Lab., 1903.

the blood, a procedure that can be done by every one. No man who has the welfare of his patients and the community at heart is ever too busy to look at a drop of blood under a microscope in his cases of continued and remittent fevers, where the diagnosis is not written all over the patient in bold medical characters. If the practitioner does not feel competent to look at the blood himself there are very few places now where there is not some one who has had microscopic training.

What can we tell from a drop of fresh blood and from a slide of stained blood that will help us to differentiate the continued fevers? First of all as we look at a drop of blood the color and size of the corpuscles tell us if there is much anemia; the anemia, however, we can see better in the visible mucous membranes of the patient. The rapidity with which crenation, endoglobular degeneration and fibrin formation occur give us some idea of the state of the blood. A little practice soon enables one to form accurate judgment of the value of these changes; most important of all, we can see whether or not the malarial parasite is present. Here let me sound a note of warning. One examination is not always sufficient to exclude malaria, although if several smears of blood are examined and no organisms are seen it generally will exclude a malarial infection. However, to be certain, one must withhold quinine and examine for several successive days. Again, if much quinine has been given, the parasites may disappear from the circulating blood completely, and our means of diagnosis is taken from us. It is best in cases of doubt to give strychnine, and should a chill occur give a hypodermic of morphine, which quickly relieves the patient but has no effect on the sporulation of the malarial organisms. However, in the estivo-autumnal form, the form that simulates typhoid fever, the parasites can be found in the peripheral circulation some days after even large doses of quinine. Especially is this the case if ovoids and crescents, the forms destined to propagate the parasite in the body of the mosquito, are present.

But to return, no parasites mean no malaria. One swallow does not make a summer, but one malarial organism seen in a drop of blood from a patient with fever makes malaria.

Then we can tell from the fresh specimen if there is a marked increase in the number of the leucocytes. Two to three white cells seen in every field of a one twelfth inch oil immersion is a leucocytosis of 12,000 to 14,000. Such a condition would either indicate endocarditis, septicemia, possibly a pneumonia, or would indicate a complication, usually septic, in a typhoid fever or malaria. A low leucocyte count is found in both uncomplicated typhoid fever and malaria. A large number of white cells with highly refractile granules and active ameboid movements, eosinophiles, would cause one to suspect trichiniasis or uncinariasis. Cases of the former simulating typhoid fever have been reported by Osler (*Amer. Jour. Med. Sc.*, March, 1899), and McCrae has put on record a case (*Ibid.*, July, 1902) where trichiniasis and typhoid fever occurred in the same patient. Whether or not uncinariasis simulates typhoid or malaria I do not know, but I think it a possibility. A microscopic examination of the stools in a suspected case would soon settle the diagnosis.

What can we tell from a stained specimen? Really, very little that we can't see in a fresh specimen except the relative numbers of the different varieties of white cells and the presence of such pathological elements as nucleated red corpuscles and myelocytes. These are best calculated by making a count, by means of the mechanical stage, of at least 500 white cells, and obtaining the percentage of every variety. However, a little practice will soon enable one to tell if any one kind of white cell is markedly increased or decreased. The presence of a large number of large mononuclear cells in a normal or subnormal leucocyte count would speak for malaria, although the differential count in the two conditions is not of any great value.*

To recapitulate, in a fresh specimen of blood we can tell the following: (1) General condition of the blood-cells, anemia, etc.; (2) Leucocytosis; (3) Eosinophilia, or marked increase in any of the

---

*Higley—Differential Leucocyte Count in the Early Days of Typhoid Fever. *Med. Rec.*, September 19, 1903.

Poech—Ueber das Verhalten der Weissen Blutkërperchen bei Malaria. *Zeitsch. f. Hygiene*, Bd. XLII, 1903, pp. 563–626.

Delany—The Diagnostic Value of the Blood Counts in Malaria and Other Fevers. *Brit. Med. Jour.*, March 28, 1903.

Rogers—The Differentiation of the Continued and Remittent Fevers of the Tropics by the Blood Changes. *Lancet*, May 30, 1903.

white cells; (4) Presence or absence of malarial organisms. In a stained specimen we can see all the conditions above enumerated, besides enabling us to make a differential count of the white cells and to note the presence of pathological cells, such as nucleated red cells and myelocytes. It is sometimes easier to take a smear and stain it later and examine it at one's leisure. Wright's stain (*Jour. Med. Research,* vol. 2, 1902, p. 138) in the writer's hands has proved the best and most rapid method. No preliminary fixation is necessary and the whole procedure takes only five minutes.

Finally let us be accurate in our diagnosis, for on accurate diagnosis depends intelligent treatment. In the physician's armamentarium there are comparatively few drugs of whose action he is certain, but he does know that quinine is a specific for malaria, whereas it has not the slightest effect on the course of typhoid fever. The microscope and a drop of blood is all that is necessary in the great majority of cases to clear up the diagnosis of doubtful continued and remittent fevers. A protracted fever in our country occurring in the autumn that resists quinine and shows no malarial parasites in the circulating blood is unquestionably in most cases typhoid fever.

Let us then use the microscope constantly and persistently. This valuable clinical aid is easily accessible to all and will tend to make us more and more skeptical of typho-malaria, and more and more careful in the general examinations of our patients and more certain in our diagnosis. Moreover it will help to lift the veil of obscurity and doubt that now exists with regard to our protracted fevers, and help to put the classification of these fevers on a sound and scientific basis.

*123 Liberty Street E.*

